# Quantitative ASL Perfusion and Vessel Wall MRI in Tuberculous Meningitis: A Pre- and Post-Treatment Study

**DOI:** 10.3390/jcm15020424

**Published:** 2026-01-06

**Authors:** Yilin Wang, Zexuan Xu, Dong Xu, Dailun Hou

**Affiliations:** 1 Department of Radiology, Beijing Tuberculosis and Thoracic Tumor Research Institute, Beijing 101149, China; wangyilin_yx@163.com; 2 Department of Radiology, Beijing Chest Hospital, Capital Medical University, Beijing 101149, China; xvzx0622@163.com (Z.X.); xudong1913@sina.com (D.X.)

**Keywords:** tuberculous meningitis, infarction, arterial spin labeling, vasculitis, cerebral perfusion

## Abstract

**Background:** Tuberculous meningitis (TBM) is a severe central nervous system infection that can lead to cerebral vasculitis and infarction. This study aimed to evaluate changes in cerebral perfusion and vasculitis on magnetic resonance imaging (MRI) before and after anti-tuberculosis treatment, focusing on both infarcted and non-infarcted brain regions and comparing them with age-matched controls. **Methods:** Quantitative arterial spin labeling (ASL) perfusion and black-blood vessel wall MRI were performed at diagnosis and after 3–6 months of treatment in TBM patients and healthy controls. Regions of interest included infarcted areas, the contralateral normal brain, and TBM-affected regions without infarction. Cerebral blood flow (CBF), perfusion grading, and vasculitis were assessed and correlated with clinical stage and disease severity. **Results:** In total, 73 TBM patients and 26 controls were included. Among the patients, 26 (35.6%) had acute infarctions, mainly in the basal ganglia and corona radiata, and 65 (89.0%) exhibited vasculitis predominantly involving anterior circulation. Pretreatment MRI showed significantly reduced CBF in infarcted regions compared with contralateral brain and controls (*p* < 0.05), and both contralateral and non-infarcted TBM regions also showed lower CBF than controls (*p* < 0.05). After treatment, CBF increased significantly in non-infarcted regions (*p* < 0.05), and post-treatment perfusion grade correlated with TBM stage and vasculitis severity. **Conclusions:** TBM-related infarcts demonstrated marked hypoperfusion, while non-infarcted regions exhibited reversible ischemic changes. ASL and vessel wall imaging can quantitatively monitor treatment response and vascular inflammation, as well as predict late infarction in TBM patients.

## 1. Introduction

Tuberculosis (TB) remains a major infectious disease worldwide, with high morbidity and mortality rates, particularly in developing countries [1]. Tuberculous meningitis (TBM) is the deadliest form of extrapulmonary TB, accounting for 10–15% of all extrapulmonary manifestations and is associated with poor neurological outcomes [2,3,4]. Mycobacterium tuberculosis spreads to the meninges and brain parenchyma via the hematogenous route, forming multiple tuberculous granulomas in subcortical locations whose rupture releases bacilli into the cerebrospinal fluid (CSF), leading to TBM. The hallmarks of TBM include meningeal inflammation and thick gelatinous exudate at the base of the brain [5,6]. Cerebral infarction is a common complication, affecting up to two-thirds of patients and contributing to poor prognosis [7,8].

The blood–brain barrier (BBB) is a critical interface between the vasculature and neuronal tissues, consisting predominantly of endothelial cells connected by tight junctions [9]. It maintains central nervous system (CNS) homeostasis by regulating molecular transport and protecting the brain from toxins, with BBB disruption implicated in various neurological disorders, including infections, tumors, trauma, and stroke [10]. In TBM, inflammatory cytokines, chemokines, and pathogen-related factors compromise BBB integrity [8,11].

Conventional magnetic resonance imaging (MRI) can detect leptomeningeal enhancement and intracranial complications [12,13], but quantitative assessment of subtle BBB disruption has historically been challenging. Dynamic contrast-enhanced (DCE) MRI has been employed to measure BBB integrity, yet it requires exogenous contrast agents, which may limit repeatability and safety.

Arterial spin labeling (ASL) is a fully non-invasive MRI technique that measures cerebral blood flow (CBF) using magnetically labeled arterial water [14]. ASL can detect subtle changes in perfusion related to BBB dysfunction, as water molecules cross the BBB via both passive and active transport mechanisms. Compared with gadolinium-based methods, ASL allows absolute quantification of CBF without the need for exogenous contrast, making it suitable for the longitudinal monitoring of TBM patients.

TBM-associated exudates are predominantly located along basal brain surfaces and adjacent interstitial spaces. Early pathological studies revealed intracranial vasculitis involving vessels near tuberculous lesions, affecting proximal arteries more than leptomeningeal vessels [15], and autopsy studies confirmed extensive vasculitis involving both proximal arteries and distal perforators of the Circle of Willis [15].

Vascular wall imaging (VWI) is a relatively recent MRI technique that evaluates vessel wall structure, plaque characteristics, and inflammatory changes [16,17]. Though it can identify concentric wall enhancement indicative of vasculitis, VWI’s application in TBM is limited. Currently, ASL perfusion imaging has not been systematically applied to cerebral perfusion assessment in TBM, and combined evaluation with VWI remains unexplored.

The diagnosis of CNS vasculitis in TBM remains challenging. While histopathological confirmation is the diagnostic gold standard, brain biopsy is invasive and suffers from sampling error, particularly when inflammation affects proximal large arteries. Consequently, clinical practice increasingly relies on a supportive clinico-radiologic pattern. In this context, high-resolution MRI, especially black-blood VWI with contrast enhancement, has emerged as a pivotal non-invasive tool for detecting vessel wall inflammation and stenosis.

This study aimed to evaluate cerebral perfusion and vasculitis in TBM patients using ASL and black-blood VWI, working to examine the relationships between perfusion deficits, vasculitis, and clinical severity before and after antituberculosis treatment.

## 2. Materials and Methods

### 2.1. Patients

This prospective study included 73 patients with clinically diagnosed TBM at Beijing Chest Hospital, recruited from November 2023 to October 2024. Twenty-six healthy volunteers served as age-matched controls. The study was approved by the Ethics Committee of Beijing Chest Hospital (BJXK-2024-KY-16), and all procedures complied with institutional and national ethical standards, including the 1964 Helsinki Declaration. Written informed consent was obtained from all participants. TBM was defined as “definite” when confirmed by CSF AFB smear, culture, or PCR [18]; “probable” if patients had active pulmonary TB on chest X-ray, positive AFB in non-CSF samples, or other evidence of extrapulmonary TB [18]; and “possible” if ≥4 of the following criteria were met: TB history, lymphocyte-predominant CSF pleocytosis, illness > 5 days, CSF/plasma glucose < 0.5, absence of Cryptococcus, altered mentation, turbid CSF, or focal neurological signs [18]. Level of consciousness was assessed using the Glasgow Coma Scale (GCS). Patient demographics, clinical features, CSF analyses, and serological tests (hyponatremia, CRP, sedimentation, D2 aggregates, BNP) were recorded, and TBM severity was graded by modified British Medical Research Council criteria [19].

Participants were ≥16 years old, newly diagnosed, and without prior meningitis or neurological deficits. Patients with known co-infection of human immunodeficiency virus (HIV) were excluded in order to specifically evaluate TBM-related vasculitis. Exclusion criteria included cerebrovascular disease, epilepsy, brain injury or surgery, brain tumors, CNS disorders, pregnancy, and MRI contraindications. All patients received standardized antituberculosis therapy (9–18 months) with an intensive phase of ethambutol, isoniazid, rifampicin, and pyrazinamide, followed by maintenance therapy, and severe cases received steroids. Patients were followed-up with at 3–6 months.

### 2.2. Imaging Protocol

All patients underwent MRI on a 3T Signa Architect (GE Healthcare, Chicago, IL, USA) with a 48-channel array head coil. Before the MRI examination, the examiner and the family signed an informed consent form for the MRI examination. The scans consisted of our institution’s standard protocol for patients with TBM, including axial and sagittal T2-weighted, axial T1-weighted, axial T2-FLAIR, axial diffusion-weighted, postcontrast axial and sagittal T1-weighted, and coronal HS 3D CUBE T1WI with FS imaging, ASL, and a coronal high-resolution three-dimensional variable-flip-angle turbo spin echo T1-weighted imaging (3D CUBE T1WI) sequence with fat suppression (FS). For the present study, only ASL and CUBE T1WI images were assessed; the remaining standard protocol sequences are not further discussed.

Fat-suppressed 3D Cube T1WI was also performed (TR = 600 ms, TE = 14 ms, FOV = 20 cm; acquisition matrix (frequency × phase) = 260 × 260, slice thickness= 0.8 mm, slices = 128, ETL = 24; number of excitations = 1, total scan time = 3 min 51 s). Postcontrast sequences were performed 2 min after injecting gadobenate dimeglumine (MultiHance; Bracco) at 0.1 mmol/kg (0.2 mL/kg), with conventional 2D T1WI acquired immediately after 3D Cube T1WI. In this study, 3D black-blood CUBE flowing blood displayed a low signal due to the flow-void effect, and stationary hydrogen protons had a relatively high signal. We employed an isotropic scanning technique which enables 3D and thin-layer reconstruction in any direction after scanning, thereby improving the spatial resolution of 3D black-blood imaging with whole-brain coverage within an appropriate scanning time. This facilitates a more accurate assessment of the canal wall field [16,17].

#### 2.2.1. Image Processing and Perfusion Analysis

**Figure 1:**
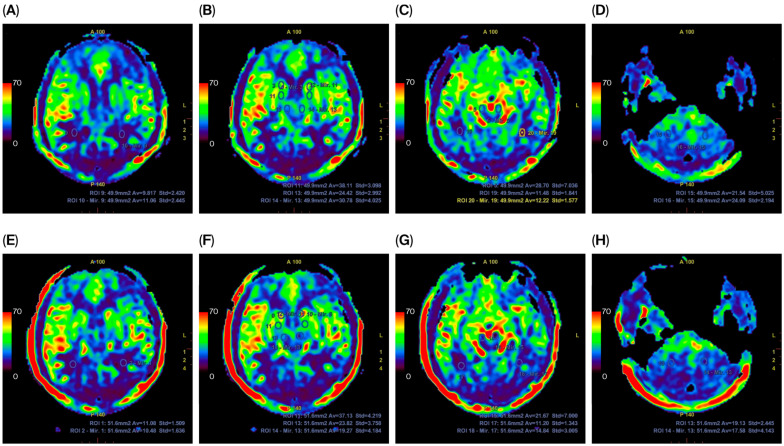
The top row represents pretreatment MRI perfusion (CBF) maps (**A**–**D**), and the bottom row represents post-treatment MRI perfusion (CBF) maps (**E**–**H**), showing a general increase in CBF. The blue and red areas (bottom and top of the color scale, respectively) indicate the lowest and highest perfusion values, respectively.

The images were reviewed by two neuroradiologists (with 15 and 7 years of experience, respectively) through consensus using AW 4.7. Infarction location and the extent of vasculitis were documented. MRI perfusion was analyzed using cranial perfusion ASL software (v 15.0), which generated parametric maps, and regions of interest (ROIs) were positioned in infarcted areas and mirrored contralaterally to determine relative CBF (Figure 1). In cases of TBM without infarction, ROIs were positioned in the basal ganglia, thalamus, internal capsule, corona radiata, occipital lobe, midbrain, and cerebellum, while for normal control cases, they were placed in the bilateral basal ganglia, thalamus, internal capsule, corona radiata, and occipital lobe. Perfusion parameters at diagnosis were compared with those at 3–6-month follow-up and graded accordingly, with measurements repeated twice and averaged. Perfusion changes were graded as follows: Grade -1 (<−5 mL/100 g/min, deteriorated), Grade 0 (−5–5, unchanged), Grade 1 (5–20), Grade 2 (20–35), and Grade 3 (>35, significantly improved). Intracranial vessels (superior cerebellar artery (SCA), anterior inferior cerebellar artery (AICA), basilar artery (BA), posterior cerebral artery (PCA) P1–P2, internal carotid artery (ICA) cavernous sinus/superior cranial segment, middle cerebral artery (MCA) M1–M4, anterior cerebral artery (ACA) A1–A2) were assessed using black-blood imaging; concentric wall enhancement indicated vasculitis. Findings at diagnosis were compared with those at 3–6-month follow-up.

#### 2.2.2. Statistics

Data were analyzed using SPSS 26.0 and Prism 10.31. Normality assessment revealed that most data, including cerebral blood flow (CBF), did not meet the assumption of normal distribution; therefore, nonparametric tests were employed. Categorical data were analyzed using Fisher’s exact or the Chi-square test, and continuous data were presented as median (IQR). For univariate analysis, comparisons between two independent groups were performed with the Wilcoxon rank-sum test, while paired pre- and post-treatment comparisons were conducted with the Wilcoxon signed-rank test. Multivariate analysis was performed using logistic regression. Statistical significance was defined as *p* < 0.05.

## 3. Results

### 3.1. Clinical Baseline Information

Seventy-three patients meeting the diagnostic criteria for TBM were enrolled. Of these, 20 (27%) completed follow-up MRI at 3–6 months, while the remaining discontinued for various reasons. Forty-four (60%) patients were male, and the mean age was 45.7 ± 15.8 years. The age distribution was as follows: 16–19 years, 4 (5.5%); 20–29 years, 11 (15.1%); 30–39 years, 9 (12.3%); 40–49 years, 18 (24.7%); 50–59 years, 17 (23.3%); 60–69 years, 8 (11.0%); and 70–79 years, 6 (8.2%). Among the 26 age-matched controls, 12 (46%) were male, with comparable age distribution (Table 1a).

Regarding disease severity, 50 (68.5%) patients were classified as stage I, 16 (21.9%) as stage II, and 7 (9.6%) as stage III, with 33 (45.2%) cases being “definite,” 34 (46.6%) “probable,” and 6 (8.2%) “possible” TBM. Univariate analysis showed that fever, vomiting, and elevated C-reactive protein were significantly associated with cerebral infarction (*p* < 0.05; Table 1a), whereas no clinical variables were significantly associated with vasculitis (Table 1b).

Furthermore, it is crucial to consider HIV co-infection as a significant confounding factor in studies of cerebral vasculitis, as HIV itself can lead to a cerebral angiitis phenotype that may mimic or complicate the radiographic presentation of TBM-associated vasculitis [20]. In our study, this potential confounder was mitigated by the exclusion of HIV-positive individuals, thereby strengthening the assertion that the vascular pathologies observed in our cohort are attributable to tuberculosis.

### 3.2. Frequency of Cerebral Infarction

Acute cerebral infarction was identified in 26 (35.6%) patients via MRI, with a total of 48 distinct infarct lesions observed. Focal neurological deficits occurred in 28 (38.4%) patients, with unilateral and bilateral infarctions in 37.5% and 16.7%, respectively. One (3.8%) patient exhibited nearly symmetrical bilateral infarctions.

Infarctions predominantly involved the anterior circulation (13/26, 50.0%) (Table 2a), with the most frequent sites being the corona radiata (57.7%), basal ganglia (34.6%), and corpus callosum (23.1%) (Table 2b). Among patients who underwent repeat MRI (n = 20), new parenchymal infarcts were observed in two cases (10.0%).

### 3.3. Vessel Wall Abnormalities on Black-Blood Imaging

At baseline, concentric vessel wall enhancement on black-blood MRI was observed in 65 (89.0%) patients, predominantly in the anterior circulation. Posterior circulation involvement coexisted in 51 (78.5%) cases. The most frequently affected segments were the M1 of the MCA (40/130 segments, 30.8%), P1 of the PCA (41/130, 31.5%), and the intracranial ICA (42/65, 64.6%). The A1 segment of the ACA was involved in 27 (41.5%) patients, whereas A2 was least affected (10/65, 15.4%).

Posterior circulation vasculitis primarily involved the PCA, with nine (13.8%) patients showing multi-segment (P1–P3) enhancement. Only 1 (1.5%) patient with infarction lacked vasculitic changes, whereas 40 (61.5%) patients with vasculitis showed no infarction.

### 3.4. Follow-Up

Follow-up MRI was scheduled and performed after 3 to 6 months of anti-tuberculous treatment (mean ± standard deviation: 4.3 ± 0.9 months; median: 4.1 months), chosen to assess early treatment response while allowing for practical patient scheduling.

Among the 20 patients who underwent repeat MRI at 3–6 months, the mean age was 44.4 ± 10.7 years and 11 were male. In this group, 95% had definite/probable TBM, 50% had cerebral infarction on baseline MRI, and 85% showed vasculitis on baseline VWI. These characteristics did not differ significantly from those of the 45 patients who did not undergo follow-up MRI (all *p* > 0.05, see Supplementary Table S1), indicating the representativeness of the follow-up cohort.

Among the 20 followed-up patients, 17 had vasculitis. The remaining three patients in the follow-up cohort had no evidence of vasculitis on either baseline or follow-up MRI, demonstrating stability in their vascular imaging phenotype; Table S3 shows the detailed clinical information of the three patients. While no patients showed complete regression of vascular changes, six (30.0%) achieved partial remission, five (25.0%) demonstrated radiologic progression, two (10.0%) showed mixed improvement and deterioration, and four (20.0%) remained stable. The specific intracranial vascular segments that demonstrated new involvement or progression in these 5 patients are detailed in Supplementary Table S2.

While no fatal outcomes occurred, three patients (15.0%) developed neurological sequelae. Table 2c compares the frequency of segmental involvement between baseline and follow-up, and analysis of individual trajectories reveals heterogeneous patterns.

### 3.5. MRI Perfusion Performance

#### 3.5.1. Baseline Perfusion Findings

Perfusion analysis was performed in all 73 patients with TBM at admission, including 26 with infarction, and the 20 who underwent repeat MRI, of whom 10 showed infarction.

At baseline, ASL demonstrated significantly reduced CBF in infarcted regions compared with contralateral normal brain (22.0 vs. 36.4 mL/100 g/min, *p* < 0.05) and age-matched controls (22.0 vs. 45.2 mL/100 g/min, *p* < 0.05) (Table 3a). Although contralateral regions showed no diffusion abnormalities on DWI, their CBF was still significantly lower than that of controls (36.4 vs. 45.2 mL/100 g/min, *p* < 0.05).

**Figure 2:**
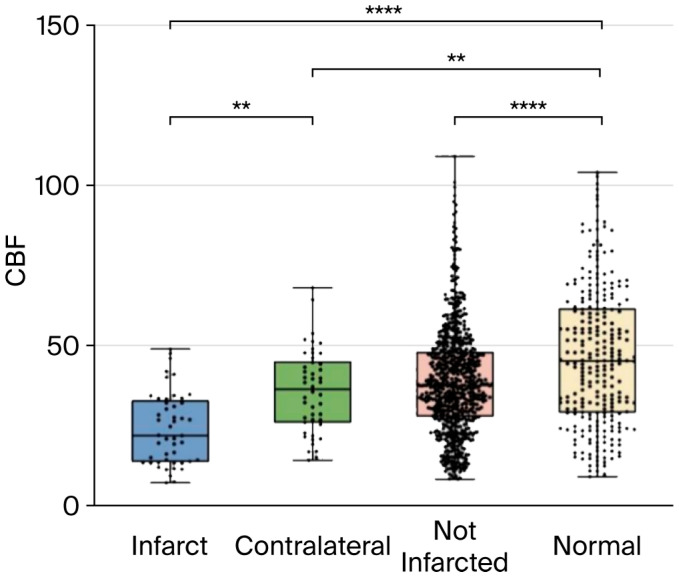
Boxplot representing pretreatment CBF perfusion parameters and significant differences between infarcted, contralateral normal, TBM without infarct, and normal, age-matched control brain. ANOVA with Šídák’s multiple comparisons test was used. ** *p* < 0.01, **** *p* < 0.0001.

Similarly, TBM patients without infarction exhibited reduced CBF compared with controls (38.0 vs. 45.2 mL/100 g/min, *p* < 0.05), suggesting subclinical hypoperfusion (Table 3b). Boxplots of pretreatment CBF values for infarcted, contralateral, and non-infarcted TBM brains compared with controls are shown in Figure 2.

#### 3.5.2. Post-Treatment Perfusion Changes

After antituberculosis therapy, CBF significantly increased in infarcted regions (22.0 → 32.6 mL/100 g/min, *p* < 0.05), contralateral normal brain tissue (35.5 → 40.3 mL/100 g/min, *p* < 0.05), and non-infarcted TBM patients (39.1 → 42.9 mL/100 g/min, *p* < 0.05) (Table 3c).

Correlations between perfusion grading, TBM stage, Glasgow Coma Scale (GCS) score, and vasculitis status before and after treatment are summarized in Table 4. Among non-infarcted TBM patients, perfusion grade was significantly associated with TBM stage and vasculitis activity before and after treatment (Table 4c). However, no significant associations were observed between perfusion grade, clinical stage, or vasculitis in infarcted or contralateral regions (Table 4a,b).

**Figure 3:**
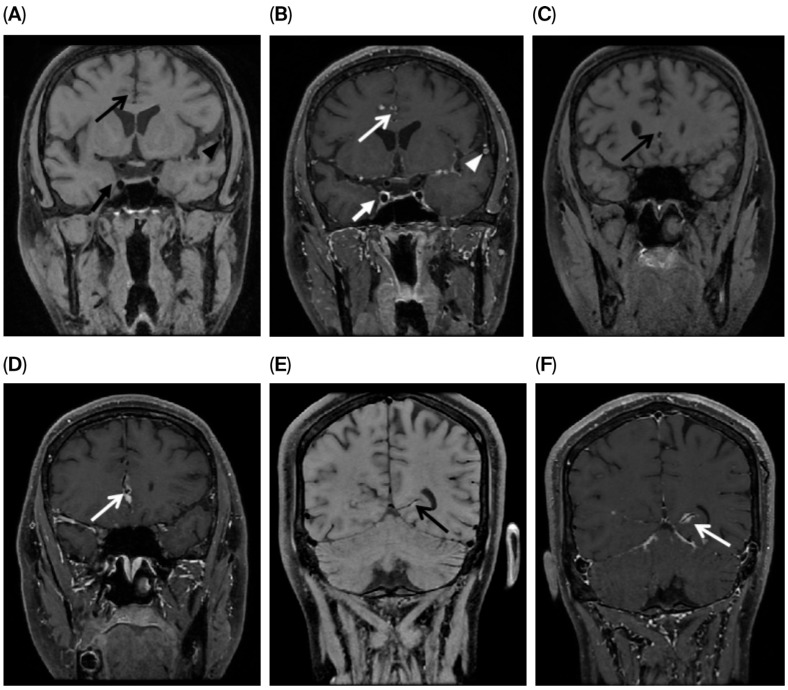
A 29-year-old male with tuberculous meningitis at first presentation. Precontrast (**A**,**C**,**E**) and postcontrast (**B**,**D**,**F**) black-blood CUBE T1 sequences (vessel wall imaging) demonstrating concentric wall enhancement in the anterior cerebral artery (long white arrow in (**B**,**D**)), right internal carotid artery (short white arrow in (**B**)), left middle cerebral artery (white triangle in (**B**)), and left posterior cerebral artery (long white arrow in (**F**)) is suggestive of vasculitis.

Longitudinal changes in perfusion grade and vasculitis status across patient subgroups are detailed in Table 5. Notably, a mild correlation was found between worsening perfusion grade and disease progression after treatment (*p* = 0.005), suggesting that the proposed perfusion grading system may serve as a predictor of delayed infarction in TBM. Representative ASL perfusion maps before and after therapy are presented in Figure 3.

## 4. Discussion

In this study, we quantitatively and qualitatively evaluated cerebral perfusion and intracranial vasculitis in patients with TBM before and after treatment using ASL and black-blood vessel wall imaging. We observed significantly reduced CBF in infarcted regions compared with both contralateral and age-matched normal brains; furthermore, contralateral regions without visible infarction, as well as TBM patients without infarcts, demonstrated subclinical ischemic changes characterized by decreased CBF.

Our findings also showed that vasculitic involvement was universal among TBM patients, predominantly affecting the anterior circulation. The distribution and severity of vasculitis closely paralleled the extent of cerebral infarction, suggesting a mechanistic relationship between vascular inflammation and ischemic injury. After treatment, cerebral perfusion improved across all patient groups. Moreover, perfusion grading in non-infarcted TBM patients correlated with vasculitis activity, underscoring the potential pathophysiological link between progressive vasculitis, impaired cerebral perfusion, and subsequent stroke risk in TBM.

The BBB serves as both a physical and functional interface between the cerebral vasculature and neural parenchyma, maintaining CNS homeostasis by tightly regulating molecular exchange between the circulation and brain tissue. Structurally, the BBB is primarily composed of endothelial cells interconnected by tight junctions, which form the selectively permeable barriers of cerebral capillaries [9].

BBB integrity can be compromised under a variety of pathological conditions, including infection, tumor, trauma, and ischemic injury. In infectious diseases such as TBM, proinflammatory cytokines and chemokines—particularly tumor necrosis factor-α (TNF-α), interleukin (IL)-1β, IL-6, and IL-8—trigger immune activation and induce the expression of matrix metalloproteinases (MMPs), which degrade tight-junction proteins and basement membranes, thereby disrupting BBB structure and function. Using dynamic contrast-enhanced MRI (DCE-MRI), Haris et al. demonstrated a significant correlation between the transfer constant (Ktrans) and MMP activity, supporting the notion that inflammatory BBB degradation contributes to altered vascular permeability and cerebral perfusion abnormalities in TBM.

Changes in MMP expression are closely associated with BBB disruption in TBM, and both animal and human studies using perfusion MRI parameters such as Ktrans have provided valuable insight into the spatial distribution of contrast agents in pathological tissues, reflecting alterations in tissue microstructure and BBB integrity [21]. However, few studies have specifically investigated the utility of perfusion MRI for assessing BBB integrity under infectious conditions [22,23].

To date, ASL has not been widely applied in TBM. In this study, we quantitatively assessed cerebral perfusion in TBM patients with infarction, contralateral normal hemispheres, non-infarct TBM, and healthy controls using ASL-MRI. Our results demonstrated a significant reduction in CBF within infarcted regions compared with both age-matched controls and contralateral hemispheres. Moreover, TBM patients without infarction also exhibited ischemic alterations and significantly reduced CBF compared with healthy controls.

During follow-up, post-treatment ASL scans revealed a significant increase in CBF across all TBM groups, indicating improved BBB permeability and cerebral perfusion. Importantly, this study is the first to propose a perfusion grading system to evaluate treatment response in TBM. The grading of perfusion improvement was significantly correlated with the coexistence of infarction, suggesting that infarcts may delay the recovery of cerebral perfusion. Collectively, our findings highlight ASL-MRI as a valuable, non-invasive technique for longitudinal assessment of cerebral perfusion in TBM without exposure to ionizing radiation or exogenous contrast agents.

TBM typically presents with mild meningeal inflammation [24]. Neuroimaging often demonstrates basal cisternal meningeal enhancement, sometimes accompanied by obstructive hydrocephalus due to thick exudates at the skull base, which impede cerebrospinal fluid circulation [25]. Cerebral dysfunction may result from small-vessel vasculitis secondary to direct bacterial invasion or immune-mediated injury [26]. Cerebral infarctions in TBM most frequently occur within the medial striate and thalamoperforating arteries, whereas lateral striate, anterior choroidal, and thalamic or cortical branch involvement is less common [26,27]. Globally, stroke incidence among TBM patients is approximately 30%, making it one of the most common and devastating complications [28]. Misra et al. reported that cerebral ischemia occurs in 13–57% of TBM cases [29] due to underlying mechanisms including arteritis, vasospasm, and arterial thrombosis [30].

MRA typically demonstrates segmental stenosis, a beaded appearance, or complete occlusion of small or large intracranial arteries [26]. In this setting, VWI offers a clear advantage, as it can reveal inflammatory wall thickening before luminal narrowing becomes apparent [31,32]. In the present study, 89% of patients exhibited abnormal VWI findings, consistent with recent evidence suggesting that arterial involvement in TBM is more extensive than previously recognized [33,34]. Vessel wall enhancement typically manifests with a concentric ring-like presentation, reflecting inflammatory changes, and may even appear in patients without overt infarction [35,36,37]. Notably, intracranial vessel wall enhancement was more common than cerebral infarction, a finding corroborated by prior studies [38,39].

The arteries most frequently affected in our cohort were the intracranial segment of the ICA, the PCA’s P1 segment, and the MCA’s M1 segment, consistent with earlier findings [33,36,40]. TBM-associated vasculitis is a recognized cause of secondary infarction [41], and autopsy studies have described extensive vasculitic changes in TBM, which can be categorized as necrotizing, infiltrative, or proliferative [15,42]. Necrotizing and infiltrative lesions typically occur early, whereas proliferative lesions appear later, leading to luminal stenosis and chronic ischemia [40,42]. Acute infarction is usually attributed to vasospasm or thrombosis, whereas delayed infarction arises from proliferative vessel wall thickening [41,42].

The complete absence of vasculitis resolution within 3–6 months highlights the persistent nature of vascular inflammation in TBM, suggesting that it may outlast meningeal improvement. The observed heterogeneous response patterns (improvement in 38.9%, progression in 27.8%) may relate to initial severity, treatment timing, or host immunity, and short-term MRI monitoring may be warranted to guide therapy adjustments.

In our cohort, among 17 patients with vasculitis on follow-up, 5 demonstrated lesion progression or new vascular involvement, 6 showed improvements, 4 exhibited no significant change, 2 displayed mixed local progression and improvement, and 2 developed new infarctions (see detailed segmental progression data in Supplementary Table S1). Both patients who developed new infarcts during follow-up belonged to the subgroup with pre-existing infarction at baseline, underscoring the high-risk nature of this population.

Both acute and chronic infarcts were associated with vasculitis affecting proximal large vessels supplying the corresponding territories. Moreover, in patients without infarction, a significant correlation was observed between perfusion grade and vasculitis before and after treatment—an association not previously described. No significant correlation was identified between perfusion grade and vasculitis in infarcted or contralateral regions, likely due to the small sample size (~12% of the total ROI measurements).

Collectively, these findings suggest that intracranial black-blood VWI not only facilitates the identification of tuberculous vasculitis and its link to delayed infarction but also serves as a valuable imaging biomarker for evaluating therapeutic response and guiding the management of vascular complications in TBM.

The occurrence of new cerebral infarcts in 10% of our followed-up patients, despite ongoing antituberculosis therapy, is a concerning finding, underscoring the persistent risk of stroke in TBM. Crucially, these new infarcts developed in territories supplied by arteries that showed active, unresolved vasculitis on follow-up imaging. This strongly suggests that the angiographic (or vessel wall) response lags substantially behind the clinical and microbiological response. The inflammatory vasculopathy may enter a smoldering, proliferative phase that continues to threaten the vessel lumen through mechanisms such as progressive wall thickening, superimposed thrombosis, or vasospasm, even as the disease’s meningitic component is being treated. Therefore, the lack of rapid radiologic improvement in vasculitis does not equate to treatment failure in a bacteriological sense, but it does signal ongoing, high-risk vascular pathology that may require adjunctive therapies (e.g., corticosteroids, antiplatelet agents) or closer monitoring beyond the standard anti-tuberculous regimen.

In this study, we focused on high-resolution VWI as a more direct and potentially earlier marker of inflammatory vasculitis than lumenography techniques like MRA. While MRA demonstrates downstream luminal sequelae such as stenosis or beading, VWI can detect primary inflammatory wall thickening and enhancement that may precede significant luminal compromise. Our findings of prevalent vessel wall enhancement (89.0%) that often occurred without infarction highlight the potential of VWI for early diagnosis and risk stratification, enabling timely intervention before the development of irreversible luminal stenosis or stroke.

We also assessed the clinical features of TBM patients with cerebral infarction and vasculitis. Stroke in TBM may be asymptomatic or present as a silent event. In our cohort, fever and headache were the most common symptoms, consistent with previous studies [42], and fever and vomiting were significantly more frequent in TBM patients with infarction than in those without. Altered consciousness was observed in 19.4% of patients, within the previously reported range of 17–69% [42,43], while focal neurological deficits were present in 38.4% of cases, consistent with prior findings [8,44,45]. Cranial nerve palsy was also common, possibly due to increased intracranial pressure or basal exudates [46], including cranial neuropathy, which may be caused by vasculitis or inflammation, is seen in 17–70% of patients with CNS tuberculosis, and cranial nerve enhancement can be depicted. Which may contribute to strokes in the basal ganglia region supplied by the medial and lateral striate and thalamic perforating arteries [42,47]. This pattern aligns with our findings that infarcts most often occurred in the basal ganglia and internal capsule.

This study has several limitations. First, the rate of complete longitudinal MRI follow-up at 3–6 months was low (27%). This was primarily due to financial constraints for patients, logistical challenges in returning to our tertiary center, clinical decisions to forgo imaging in improving patients, and disruptions caused by the COVID-19 pandemic. While this may introduce selection bias into the analysis of imaging evolution, it is important to note that the majority of patients without MRI follow-up remained under clinical supervision to ensure treatment completion. Second, the proportions of patients with stage I–III TBM were not balanced, as most had early-stage disease. Despite these limitations, our prospective study provides new insights into the link between BBB disruption, reduced cerebral perfusion, and vasculitis in TBM.

The strengths of this study include the combined use of ASL and black-blood MRI to clarify TBM pathophysiology and assess vascular complications longitudinally, demonstrating that BBB impairment correlates with vasculitis severity, clinical stage, GCS, CSF levels, and inflammatory markers. Follow-up MRI revealed improved perfusion and vasculitis regression in some patients after 3–6 months of treatment.

## 5. Conclusions

BBB disruption in TBM is associated with cerebral hypoperfusion and infarction. ASL and black-blood imaging can detect vasculitis and monitor disease progression, and the recurrence or worsening of vascular lesions may predict delayed infarction and the need for prolonged therapy. Therefore, combining ASL with black-blood sequences may improve management and prognostic evaluation in TBM.

## Figures and Tables

**Table 1:** (**a**) Univariate analysis exploring the correlation of various clinical data, MRI manifestations, and CSF analysis with infarction in TBM patients. (**b**) Univariate analysis exploring the association of various clinical profiles, clinical stagings, and CSF parameters with vasculitis in TBM patients.

(a)				
Parameters *n* (%)	Total (*n* = 73)	Infarct (*n* = 26)	TBM Without Infarct (*n* = 47)	*p*-Value
Age mean (SD)	45.7 (15.786)	47.04 (15.288)	44.96 (16.17)	0.593
Gender male	44 (60.2)	17 (65.4)	27 (57.5)	0.507
Vasculitis	65 (89.0)	25 (96.2)	40 (85.1)	0.245
Co-morbidity				
Hypertension	10 (13.7)	2 (7.7)	8 (17.0)	0.478
Diabetes	10 (13.7)	4 (15.4)	6 (12.8)	0.152
Fever	51 (69.9)	13 (50.0)	38 (80.9)	0.006
Consciousness disorders ^a^	14 (19.2)	6 (23.1)	8 (17.0)	0.548
Headache	36 (49.3)	12 (46.2)	24 (51.1)	0.688
Vomiting	25 (34.2)	4 (15.4)	21 (44.7)	0.012
Focal neurological deficits ^b^	28 (38.4)	11 (42.3)	17 (36.2)	0.606
Cranial nerve palsy ^c^	10 (13.7)	3 (11.5)	7 (14.9)	1
Neck stiffness	10 (13.7)	1 (3.8)	9 (19.1)	0.085
Coughing	27 (37.0)	9 (34.6)	18 (38.3)	0.755
TBM staging				0.464
I	33 (45.2)	13 (50.0)	20 (42.6)	
II	34 (46.6)	10 (38.5)	24 (51.1)	
III	6 (8.2)	3 (11.5)	3 (6.4)	
GCS staging				0.278
I	50 (68.5)	16 (61.5)	34 (72.3)	
II	17 (23.3)	6 (23.1)	11 (23.4)	
III	7 (9.6)	4 (15.4)	3 (6.4)	
CSF glucose	64	25	39	0.913
Low	31 (48.4)	12 (48.0)	19 (48.7)	
High	2 (3.1)	1 (4.0)	1 (2.6)	
Normal	31 (48.4)	12 (48.0)	19 (48.7)	
CSF Cl				0.546
Low	26 (40.6)	9 (36.0)	17 (43.6)	
CSF protein				0.599
High	41 (64.1)	17 (68.0)	24 (61.5)	
Laboratory examinations				
Hyponatremia	32 (44.4)	11 (42.3)	21 (45.7)	0.784
C-reactive protein	33 (52.4)	8 (34.8)	25 (62.5)	0.034
Blood sedimentation rate	14 (53.8)	3 (33.3)	11 (64.7)	0.218
D2 polymer	27 (60.0)	10 (62.5)	17 (58.6)	0.799
B-type natriuretic peptide (BNP)	14 (58.3)	5 (55.6)	9 (60.0)	1
**(b)**				
**Parameters *n* (%)**	**Total (*n* = 73)**	**Vasculitis (*n* = 65)**	**TBM without Vasculitis (*n* = 8)**	***p*** **-Value**
Age mean (SD)	45.7 (15.8)	45.2 (16.1)	49.8 (13.0)	0.446
Gender male	44 (60.3)	41 (63.1)	3 (37.5)	0.252
Infarct	26 (35.6)	25 (38.5)	1 (12.5)	0.245
Co-morbidity				
Hypertension	10 (13.7)	10 (15.4)	0 (0.0)	0.588
Diabetes	10 (13.7)	10 (15.4)	0 (0.0)	0.588
Fever	51 (69.9)	44 (67.7)	7 (87.5)	0.421
Consciousness disorders ^a^	14 (19.2)	11 (16.9)	3 (37.5)	0.175
Headache	36 (49.3)	34 (52.3)	2 (25.0)	0.261
Vomiting	25 (34.2)	22 (33.8)	3 (37.5)	1
Focal neurological deficits ^b^	28 (38.4)	24 (36.9)	4 (50.0)	0.473
Cranial nerve palsy ^c^	10 (13.7)	9 (13.8)	1 (12.5)	1
Neck stiffness	10 (13.7)	8 (12.3)	2 (25.0)	0.3
Coughing	27 (37.0)	25 (38.5)	2 (25.0)	0.702
TBM staging				0.859
I	33 (45.2)	30 (46.2)	3 (37.5)	
II	34 (46.6)	29 (44.6)	5 (62.5)	
III	6 (8.2)	6 (9.2)	0 (0.0)	
GCS staging				0.068
I	50 (68.5)	47 (72.3)	3 (37.5)	
II	16 (21.9)	12 (18.5)	4 (50.0)	
III	7 (9.6)	6 (9.2)	1 (12.5)	
CSF glucose	64	57	7	
Low	31 (48.4)	29 (51.8)	2 (28.6)	0.545
High	2 (3.1)	2 (3.6)	0 (0.0)	
Normal	31 (48.4)	26 (46.4)	5 (71.4)	
CSF Cl				1
Low	26 (40.6)	23 (41.1)	3 (42.9)	
CSF protein				0.695
High	41 (64.1)	37 (66.1)	4 (57.1)	
Laboratory examinations				
Hyponatremia	32 (44.4)	29 (45.3)	3 (37.5)	0.725
C-reactive protein	33 (53.2)	29 (50.9)	4 (66.7)	0.674
Blood sedimentation rate	14 (53.8)	13 (56.5)	1 (33.3)	0.580
D2 polymer	27 (60.0)	21 (55.3)	6 (85.7)	0.215
B-type natriuretic peptide (BNP)	14 (58.3)	13 (61.9)	1 (33.3)	0.550

Consciousness disorders ^a^: Drowsiness, coma, stupor, blurred consciousness, delirium; focal neurological deficits ^b^: limb weakness, sensory abnormalities, speech difficulties, visual–auditory deficits; cranial nerve palsy ^c^: dizziness, coma, memory loss.

**Table 2:** (**a**) Infarct location and pattern at clinical presentation in patients with TBM; (**b**) Areas of vasculitis involvement; (**c**) Comparison of vasculitis involvement by intracranial segment before and after treatment (follow-up cohort).

(a)			
Location of Infarcts *n* (%)	Number of Patients (*n* = 26)	Pattern of Infarcts *n* (%)	Number of Patients (*n* = 26)
Basal ganglia	9 (34.6%)	Unilateral	18 (69.2%)
Internal capsule	3 (11.5%)	Bilateral	8 (30.8%)
Thalamus	3 (11.5%)	Anterior circulation	13 (50.0%)
Corona radiata	15 (57.7%)	Posterior circulation	7 (26.9%)
Occipital lob	5 (19.2%)	Anterior and posterior circulation	6 (23.1%)
Cerebellum	2 (7.7%)		
Brainstem (midbrain and pons)	5 (19.2%)		
Corpus callosum	6 (23.1%)		
**(b)**			
**Vascular Segment**		**Frequency (%)**	
ACA		31 (47.7%)	
A1		27 (20.8%)	
A2		10 (7.7%)	
MCA		62 (95.4%)	
M1		40 (30.8%)	
M2		22 (16.9%)	
M3		29 (22.3%)	
M4		35 (26.9%)	
PCA		49 (75.4%)	
P1		41 (31.5%)	
P2		26 (20.0%)	
P3		11 (8.5%)	
BA		17 (26.2%)	
ICA		42 (64.6%)	
SCA		22 (33.8%)	
AICA		11 (16.9%)	
**(c)**			
**Vascular Segment**	**Baseline Frequency (%)**	**Follow-Up Frequency (%)**	**Change (Δ)**
ACA	9 (50.0%)	7 (38.9%)	↓
A1	15 (41.7%)	11 (30.6%)	↓
A2	4 (11.1%)	0	↓
MCA	17 (94.4%)	14 (77.8%)	↓
M1	21 (58.3%)	13 (36.1%)	↓
M2	12 (33.3%)	7 (19.4%)	↓
M3	15 (41.7%)	9 (25.0%)	↓
M4	18 (50.0%)	11 (30.6%)	↓
PCA	14 (77.8%)	12 (66.7%)	↓
P1	24 (66.7%)	20 (55.6%)	↓
P2	13 (36.1%)	11 (30.6%)	↓
P3	5 (13.9%)	3 (8.3%)	↓
BA	4 (22.2%)	3 (16.7%)	↓
ICA	9 (50.0%)	8 (44.4%)	↓
SCA	6 (33.3%)	5 (27.8%)	↓
AICA	3 (16.7%)	2 (11.1%)	↓

↓ It refers to the situation where, after treatment, the accumulated vascular segments of vasculitis have decreased less than those before treatment.

**Table 3:** (**a**) Comparison of perfusion parameter medians and *p*-values between patients with cerebral infarction, contralateral normal brain, age-matched controls. (**b**) Comparison of perfusion parameters between controls, contralateral normal brain, and no-infarction TBM patients. (**c**) Pre- and post-treatment perfusion parameters of infarcted, contralateral normal, and no-infarction TBM brains.

(a)			
Perfusion Pretreatment	CBF (mL/100 g/min) Median (IQR)		*p*-Value
Infarcts (*n* = 48)	21.955 (13.565, 33.095)		
Normal-Appearing Contralateral Brain (*n* = 46)	36.375 (25.858, 45.173)		0.000
Age-Matched Controls (*n* = 260)	45.200 (29.030, 61.648)		0.000
**(b)**			
**Perfusion Pretreatment**	**CBF (mL/100 g/min) Median (IQR)**		***p*** **-Value**
Age-Matched Controls (*n* = 260)	45.2 (29.0, 61.6)		
Contralateral Normal Brain (*n* = 46)	36.4 (25.9, 45.2)		0.002
No-infarction TBM (*n* = 508)	38.0 (26.6, 48.9)		0.000
**(c)**			
**Perfusion Parameters**	**Perfusion Pretreatment Mean (IQR)**	**Post-Treatment Median (IQR)**	***p*** **-Value**
Infarcts (*n* = 23)	22.0 (14.2, 32.2)	32.6 (20.7, 40.3)	0.001
Contralateral Normal Brain (*n* = 23)	35.5 (26.8, 42.5)	40.31 (33.6, 46.6)	0.016
No-infarction TBM (*n* = 140)	39.1 (30.0, 49.1)	42.94 (33.3, 53.2)	0.014

Data for the “Infarcts” group are presented per lesion (*n* = 48 lesions from 26 patients); age-matched controls (ROI measurements, *n* = 260).

**Table 4:** Pre- and post-treatment correlation between clinical and perfusion grade in infarct-TBM (**a**) and normal contralateral brain (**b**). (**c**) Univariate analysis of clinical stage, prognostic stage, and vasculitis correlation and *p*-value with pre- and post-treatment perfusion grading for TBM without infarction.

(a)						
Perfusion Grade of Infarct-TBM	Grade −1	Grade 0	Grade 1	Grade 2	Grade 3	*p*-Value
TBM grade						0.612
I	1	4	9	2	0	
II	1	0	5	1	0	
III	0	0	0	0	0	
GCS grade						0.662
I	1	3	7	2	0	
II	1	1	2	1	0	
III	0	0	5	0	0	
Vasculitis	1	4	13	3	0	0.253
**(b)**						
**Perfusion Grade of Contralateral Normal Brain**	**Grade −1**	**Grade 0**	**Grade 1**	**Grade 2**	**Grade 3**	***p*** **-Value**
TBM grade						1
I	1	9	5	1	0	
II	0	4	3	0	0	
III	0	0	0	0	0	
GCS grade						0.451
I	1	9	3	0	0	
II	0	2	2	1	0	
III	0	2	3	0	0	
Vasculitis	1	13	7	0	0	0.067
**(c)**						
**Perfusion Grading of TBM without Infarction**	**Grade −1**	**Grade 0**	**Grade 1**	**Grade 2**	**Grade 3**	***p*** **-Value**
TBM Staging						0.000
I	19	23	36	6	0	
II	17	17	6	9	7	
III	0	0	0	0	0	
GCS staging						0.135
I	1	9	3	0	0	
II	0	2	2	1	0	
III	0	2	3	0	0	
Vasculitis	31	25	34	7	1	0.000

**Table 5:** Univariate analysis showing the correlation and *p*-values of perfusion with pre- and post-treatment perfusion grading and vasculitis in patients with infarction, contralateral normal brains, and TBM without infarction.

	Infarct	Normal-Appearing Contralateral Brain	TBM without Infarcts	*p*-Value
Perfusion grading				0.005
Deteriorated	2	1	36	
Unchanged	4	13	40	
Improved	17	9	57	
Significantly improved	0	0	7	
Vasculitis	9	0	8	0.074
Improved	1	0	5	
Aggravated	3	0	2	
Merged *	1	0	1	
Unchanged	4	0	0	

* Vasculitis partially aggravated and partially improved.

## Data Availability

The data used to support the findings of this study are included in the article.

## Supplementary Materials

The following supporting information can be downloaded at: https://www.mdpi.com/article/10.3390/jcm15020424/s1, Table S1: Baseline characteristics of the entire TBM cohort and the subgroup with 3-6 months. Table S2: Detailed vasculitis progression in patients with baseline vasculitis (*n* = 5). Table S3: Characteristics of the three follow-up patients without vasculitis.

## Abbreviations

TBM	Tuberculosis
MRI	Magnetic Resonance Imaging
ASL	Arterial Spin Labeling
CBF	Cerebral Blood Flow
BBB	Blood–Brain Barrier
CNS	Central Nervous System
VWI	Vascular Wall Imaging
CSF	Cerebrospinal Fluid
AFB	Acid-Fast Bacilli
PCR	Polymerase Chain Reaction
PTB	Pulmonary Tuberculosis
GCS	Glasgow Coma Scale
HS	High-Resolution
3D CUBE T1WI	Three-Dimensional Variable-Flip-Angle Turbo Spin Echo T1-Weighted Imaging
FS	Fat Suppression
ROI	Region of Interest
MMPs	Matrix Metalloproteinases
SCA	Superior Cerebellar Artery
AICA	Anterior Inferior Cerebellar artery
BA	Basilar Artery
PCA	Posterior Cerebral Artery
ICA	Inferior Carotid artery
MCA	Middle Cerebral Artery
ACA	Anterior Cerebral Artery
