# Supplementary material for: Quantitative ASL Perfusion and Vessel Wall MRI in Tuberculous Meningitis: A Pre- and Post-Treatment Study
Source: J Clin Med. 2026 Jan 6;15(2):424. doi: 10.3390/jcm15020424 (PMC12842173; doi:10.3390/jcm15020424)
Supplement: Supplementary file 1 [file jcm-15-00424-s001.zip › jcm-4010823-supplementary.pdf]

Journal of Clinical Medicine

MDPI

Supplementary Table S1: Baseline characteristics of the entire TBM cohort and the subgroup with 3-6 months MRI follow-up

Parameters n(%)	Totally (n=73)	Follow-up （n=20）	p-value
Age Mean (SD)	45.7(15.8)	44.4(10.7)	0.346
Gender Male	44（60.3）	11 (55.0)	0.671
Infarction	26（35.6）	10 (50.0)	0.242
Vasculitis	65（89.0）	18 (90.0)	0.620
TBM staging			0.287
definite	33（45.2）	13 (65.0)	
probable	34（46.6）	6 (30.0)	
possible	6（8.2）	1 (5.0)	
GCS staging			0.953
I	50（68.5）	13 (65.0)	
II	16（21.9）	5 (25.0)	
III	7（9.6）	2 (10.0)	

Supplementary Table S2: Detailed Vasculitis Progression in Patients with Baseline Vasculitis (n=5)

Patient Number	Baseline Involved Segments	New or Progressively Involved Segments at Follow-up
Patient 1	Bilateral MCA M3, M4, PCA P1, ACA	Progressed: Left MCA M4 (increased wall thickness/enhancement)
Patient 2	Left MCA M1-M4, Right MCA M1, M3, M4	Progressed: Left M1/M4, Right M1
Patient 3	Left ICA, Left MCA M3, M4, PCA P1, P2, Right MCA M2, M3, PCA P1, Left ACA	Progressed: Left PCA P2
Patient 4	Left ICA, Left MCA M1, Right PCA P1, Left SCA	Progressed: Right PCA P1; New: Left PCA P1
Patient 5	Right MCA M4, Bilateral PCA P1	New: Left MCA M4

Supplementary Table S3: Characteristics of the Three Follow-up Patients Without Vasculitis

Parameter	Patient-01	Patient-02	Patient-03
Age/Sex	43 / Male	53 / Male	59 / Female
TBM staging	Definite	Definite	Probable
MRC staging	Stage I	Stage II	Stage I
MRI Findings	Meningeal enhancement. No vasculitis. No infarction.	Meningeal enhancement. No vasculitis. No infarction.	Mild meningeal enhancement. No vasculitis. A small non-vasculitic infarctionin the right occipital lobe.

MRI follow-up (3-6 months)	No vasculitis. Meningeal inforcement reduction.	No vasculitis. Meningeal inforcement reduction.	No vasculitis. Gliotic evolution of the pre-existing infarction. No new infarction.
Clinical Symptoms	Resolution of neurological deficits and dizziness.	Alleviation of fever, headache, and drowsiness.	Alleviation of fever, headache, and vomiting.
Overall Outcome	Improved	Improved	Improved

https://doi.org/10.3390/xxxxx
